# State of the Art Review: Glucagon-Like Peptide-1 in Obesity-Related Asthma

**DOI:** 10.1007/s00408-025-00861-z

**Published:** 2025-12-08

**Authors:** Destiny R. Gomez, Isaac Swartzman, Angela Linderholm, Bethany P. Cummings, Amir A. Zeki, Krishna M. Sundar, Nicholas J. Kenyon

**Affiliations:** 1 UC Davis Lung Center, Division of Pulmonary, Critical Care, and Sleep Medicine, Department of Internal Medicine, University of California, Davis School of Medicine, Davis, CA USA; 2 Department of Internal Medicine, UC Davis School of Medicine, Sacramento, CA USA; 3 Department of Molecular Biosciences, School of Veterinary Medicine, University of California-Davis, Davis, CA USA; 4 Department of Surgery, Division of Foregut, Metabolic, and General Surgery, Center for Alimentary and Metabolic Sciences, Sleep Medicine, Department of Medicine, University of California, School of Medicine, Sacramento, CA 95817 USA

**Keywords:** Glucagon-Like peptide-1, Asthma, Obesity, Weight loss, Airway inflammation

## Abstract

Asthma is a heterogeneous condition characterized by chronic airway inflammation, airway hyperresponsiveness, and mucin hypersecretion. Obesity-related asthma is one phenotype of asthma with significant metabolic dysregulation. A complete understanding of obesity-related asthma remains elusive, but it is most often characterized by the absence of hallmark features of Type-2 (T2) high asthma, such as eosinophilia or elevated exhaled nitric oxide (NO) levels. Patients with obesity-related and T2 low asthma with or without type 2 diabetes mellitus (T2DM) experience worse clinical outcomes, including more severe acute exacerbations. Among the Food and Drug Administration (FDA) approved drug classes for T2DM, there is a growing interest in glucagon-like peptide 1 receptor agonists’ (GLP-1RAs) ability to potentially exert effects in the airway. Previous studies found that individuals with T2DM and asthma who were prescribed GLP-1RAs, had decreased asthma exacerbations and improved lung function. However, there remains a gap in understanding GLP-1RAs mechanism of action in the lung and airways to improve pulmonary function. In this review we discuss the potential mechanisms by which GLP-1RAs may impact T2 low asthma and offer a therapeutic option for this highly prevalent disorder.

## Introduction

Asthma is characterized by airway inflammation, bronchial hyperreactivity, excessive mucin production, and airway narrowing. These asthmatic features present as repeated periods of wheezing, shortness of breath, coughing, and tightness of the chest [1]. Being the most common chronic respiratory disease, asthma continues to have a significant global disease burden across the lifespan, affecting an estimated 262 million people worldwide and is the most common chronic disease among children [2]. Obesity exacerbates asthma and contributes to a distinct phenotype known as obesity-related asthma. The Centers for Disease Control and Prevention (CDC) conducted a survey during August 2021 to August 2023 and found that obesity affects 40.3% of adults [3]. It is estimated that by 2050, more than 250 million people living in the United States (US) will be overweight or obese [4]. Obesity increases the prevalence of asthma [5] and individuals that are obese account for the majority of severe or difficult-to-control asthma cases in the US [6].

Obesity-related asthma typically manifests as a conglomerate of interconnected immuno-metabolic systems and may therefore benefit from shared therapeutic interventions, such as glucagon-like peptide-1 (GLP-1) agonism. GLP-1 receptor agonists (GLP-1RAs) have been transformative in treating type-2 diabetes mellitus (T2DM), obesity, and insulin resistance [7]. Growing interest in the role of GLP-1RAs in lung biology has led to increased human clinical studies, investigating their effects in asthma and other pulmonary diseases such as chronic obstructive pulmonary disease (COPD). Previous clinical studies have found that individuals with concurrent T2DM and asthma treated with GLP-1RAs experience decreased asthma exacerbations, asthma-related hospitalizations, and overall increased asthma control [8–10]. In this review, we will provide a framework for understanding how GLP-1 may modulate airway disease, particularly in asthma.

## Asthma and Obesity

### Asthma

**Fig. 1:**
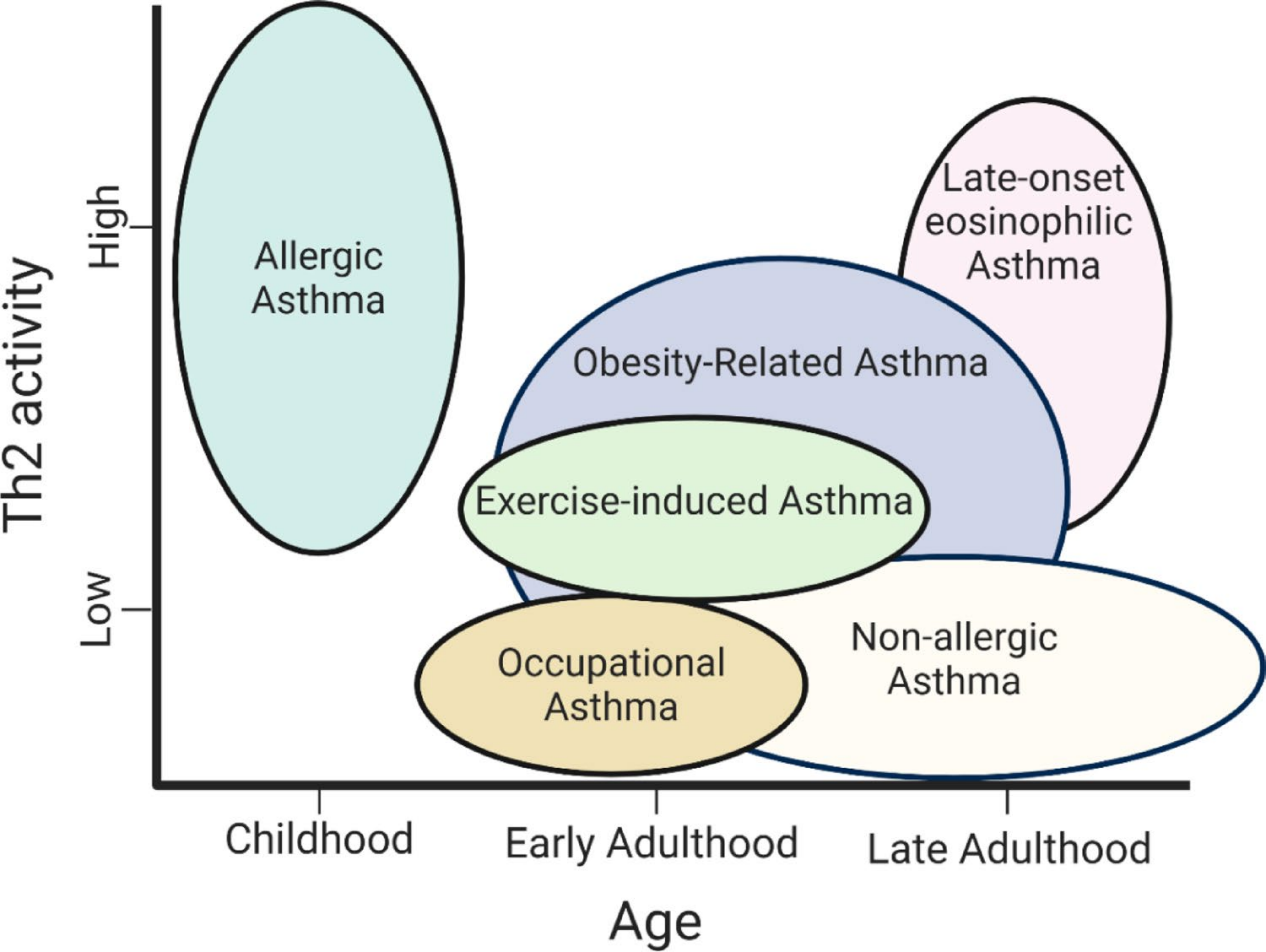
Onset of Asthma Phenotypes. Th2: T helper 2 cells

Asthma is a respiratory condition that is influenced by a genetic predisposition, and a combination of exposures including respiratory infections and environmental exposures, such as allergens and pollutants [11]. Asthma exhibits significant variability in its clinical presentation, underlying pathology, triggers, and response to treatment, posing ongoing challenges for effective treatment and disease management [12]. Asthma’s complex heterogeneity has led to the identification of distinct asthma clusters, or endotypes, that are defined by their shared clinical features, or phenotypes, shown in Fig. 1. These endotypes are often broadly grouped into eosinophilic T helper 2 inflammation (T2 high) and non-eosinophilic (T2 low) asthma. T2 high asthma is the most common, affecting primarily childhood and early adulthood, and is typically associated with eosinophilic inflammation, and is driven by cytokines, such as interleukins (IL)-4, IL-5, IL-13, which promote immunoglobulin E (IgE) production, eosinophil recruitment, and mast cell activation [13]. In contrast, T2 low asthma may involve neutrophilic or pauci-granulocytic inflammation with cytokines, such as IL-6, IL-8, IL-17 and tumor necrosis factor alpha (TNF-α), playing key roles in neutrophil recruitment and chronic inflammation. Non-allergic asthma is less well-defined in terms of biomarkers, with obesity-related asthma being frequently linked to this endotype [14–16]. These immune pathways contribute to airway hyperresponsiveness (AHR), mucin hypersecretion, and both systemic and local inflammation, ultimately leading to airflow obstruction [17, 18].

### Obesity-related Asthma

Comorbidities such as obesity, pre-diabetes and diabetes, are strong risk factors for various inflammatory diseases, including asthma [19–22]. These conditions are characterized by elevated fasting blood glucose levels, with T2DM also characterized by persistent hyperglycemia, insulin resistance, and associated systemic inflammation. Clinical studies indicate that children and adults who are overweight and obese face a heightened risk of developing asthma [23, 24]. Obesity-related asthma individuals have worsened asthma severity [25, 26], symptom burden [19, 27], the highest healthcare utilization rates [28, 29], reduced effectiveness of conventional therapies [30, 31], and different metabolic signatures [32], compared to individuals that are normal-weight and diagnosed with asthma. Obesity-related asthma individuals display low levels of exhaled nitric oxide, increased airway resistance, and diminished pulmonary function [33–35]. Interestingly, both children and adults with asthma are at an increased risk of obesity, potentially due to inactivity, corticosteroid exposure, or factors that affect both asthma and weight [36, 37]. Importantly, obesity can also worsen pre-existing T2 high asthma, highlighting that its impact is not limited to the obesity-driven asthma endotype but extends across asthma phenotypes [38]. Obesity has been observed to shift the asthmatic immune response away from the classic T2 high eosinophilic inflammation towards a T2 low phenotype, promoting a more Th1 and Th17-skewed profile, contributing to cell-mediated immunity and sustained inflammation [39]. While standard therapies, such as glucocorticoids, beta-2 adrenergic receptor (β2AR) agonists, and biologics (e.g., anti-IgE, anti-IL-5, anti-IL5R, anti-IL4R) are effective in T2 high asthma, their efficacy is often diminished or non-existent in T2 low or metabolically driven asthma phenotypes.

One of the most extensively studied mechanisms in obesity-related asthma is chronic low-grade systemic inflammation driven by excess adipose tissue. Visceral fat acts as an active endocrine organ, releasing pro-inflammatory cytokines such as IL-6, IL-8, TNF-α, and C-reactive protein (CRP), while also altering adipokine levels, marked by increased leptin and reduced adiponectin [19, 27, 31, 40]. These mediators contribute to systemic inflammation that has been strongly implicated in asthma pathophysiology [41]. Evidence suggests that excessive adiposity contributes to increased airway resistance and reduced responsiveness to corticosteroid therapy and short-acting β2AR agonists [19, 27, 31, 40]. Comorbid conditions commonly associated with obesity may further exacerbate asthma severity. For example, obstructive sleep apnea (OSA) risk has been implicated as an independent risk factor for difficult-to-control asthma, demonstrating an interplay between these two conditions [42]. Apart from shared risk factors such as obesity, gastroesophageal reflux and rhinitis, OSA contributes to intermittent hypoxia and cyclic mechanical stresses on airways increasing bronchial inflammation. This, in turn, can worsen asthma control and increase the frequency of exacerbations [43].

Beyond inflammation, obesity is linked with significant metabolic disturbances that may directly contribute to airway dysfunction. This is evident in children with obesity and asthma, where impaired glucose and insulin metabolism is associated with significantly increased AHR, both dependent and independent of BMI [44]. In a systematic review, poor glucose regulation is associated with diminished lung function, worsened asthma severity, and increased hospitalizations [45, 46]. In parallel, ex vivo studies on human bronchi have shown that hyperglycemia can directly trigger AHR. One proposed mechanism is that metabolic dysfunction impairs bronchodilation by reducing β2AR responsiveness via reduction in cyclic adenosine monophosphate (cAMP) levels [47]. Additionally, hyperinsulinemia induces a pro-contractile phenotype in airway smooth muscle (ASM) cells and increases their sensitivity to neural-mediated bronchoconstriction [48–50]. Metabolic inflammation, characterized by elevated IL-6 in the lungs and circulation has been linked to worsened asthma severity in individuals with obesity, independently of body mass index (BMI) [51]. Targeting metabolic dysfunction and systemic inflammation offers a promising therapeutic approach for improving asthma control in individuals with obesity and T2DM.

### GLP-1

#### Role of GLP-1 in Metabolic Regulation

Among the FDA-approved drug classes for T2DM and obesity, glucagon-like peptide-1 (GLP-1) receptor agonists (GLP-1RAs) have gained popularity due to their efficacy in managing obesity and diabetes. Interestingly, GLP-1RAs appear to have pleiotropic effects extending to the lungs and airway highlighting interest in their immunometabolism contribution. GLP-1 is secreted by enteroendocrine L cells, found primarily in the ileum and colon, and is released into circulation in response to food intake [52]. GLP-1 is rapidly degraded with a half-life of less than two minutes by dipeptidyl peptidase-4 (DPP-4) in humans [53]. GLP-1 increases insulin secretion in response to glucose and improves insulin sensitivity, suppresses glucagon release, and reduces appetite [7, 54]. Several studies have reported that postprandial GLP-1 secretory response from a meal to be significantly diminished in people with T2DM [55, 56]. The dual action of GLP-1 on both insulin and glucagon regulation has driven the development of pharmacological GLP-1RAs with extended half-lives, such as semaglutide, liraglutide, and tirzepatide [57]. Notably, tirzepatide is a dual incretin receptor agonist that activates both the glucose-dependent insulinotropic polypeptide (GIP) and GLP-1 receptor, distinguishing it from other GLP-1-specific agonists. Liraglutide, in particular, shares a 97% amino acid sequence with the native human GLP-1, contributing to its therapeutic effectiveness [58]. GLP-1RAs improve glycemic regulation by signaling through the GLP-1R to stimulate insulin biosynthesis and release, suppress glucagon release, delay gastric emptying, and improve glucose metabolism [59].

#### Expression of the GLP-1 Receptor

The GLP-1 receptors are expressed in multiple organs including the stomach [60], heart [61], lung [62–66], and kidney [61, 67]. Interestingly, the lung has the highest level of GLP-1 receptor mRNA expression, compared to other extra-pancreatic organs [68, 69]. The GLP-1 receptor has also been located on mouse lung epithelial and endothelial cells, and on various immune cells such as human and mouse macrophages, eosinophils, neutrophils and lymphocytes [65, 70–72]. GLP-1 receptor expression is reduced in circulating peripheral blood mononuclear cells (PBMCs) from chronic obstructive pulmonary disease (COPD) patients as compared to non-COPD controls [73]. GLP-1 concentrations are substantially higher in the bronchoalveolar lavage fluid (BALF) than in the serum [74]. In an allergic mouse model, GLP-1 levels were decreased in lung tissue and BALF following exposure to lipopolysaccharide (LPS) but were restored when treated with a GLP-1RA [75]. These studies suggest that GLP-1 may play a local immunomodulatory role in the lung, particularly in response to inflammatory stimuli.

However, the exact location of the GLP-1 receptor remains unknown, with conflicting evidence regarding its expression in the body. These disparities likely stem from difference in detection methodologies, tissue preparation techniques, and the specificity and reliability of antibodies to bind GLP-1 receptor protein in immunohistochemical analyses, highlighting the need for more standardized and sensitive approaches and better reporting in the literature [76].

## Human Clinical Studies

Growing interest in the role of GLP-1RAs in lung pathophysiology has led to increased pre-clinical animal and human clinical studies, investigating their effects in asthma and other pulmonary diseases such as COPD and OSA. There are multiple mechanisms that have been proposed for the role of GLP-1RAs on improving lung function and asthma control. We will first address how GLP-1RAs have played a role in pulmonary diseases in human clinical trials, summarized in Table 1.

**Table 1:** Human clinical studies

Reference	Study	Population	Main Findings (Comparing GLP-1 users to non-GLP-1 users)
*Observational Studies*			
Mitchell, et al. [70]	Cross-sectional study	Patients with mild allergic asthma (*n* = 10) underwent allergen inhalation challenge with peripheral blood collection vs. non-asthmatic controls (*n* = 10).	↓ Eosinophil GLP-1 receptor expression in allergic asthma patients at baseline ↓ LPS-stimulated eosinophil expression of CD69 and CD11b, ↓ Eosinophil IL-4, IL-8 and IL-13 production in mild allergic asthmatics
Rogliani et al. [78]	Prospective cohort study	Patients with T2DM (*n* = 32) treated with metformin (control cohort), metformin + GLP-1RA, or metformin + insulin	↑ FEV_1_ in GLP-1RA cohort vs. baseline; GLP-1RA cohort vs. control; GLP-1RA cohort vs. insulin cohort ↑ FVC in GLP-1RA cohort vs. control cohort; GLP-1RA cohort vs. insulin cohort ↑ MEF in GLP-1RA cohort vs. control cohort; GLP-1RA cohort vs. insulin cohort
Albogami et al. [9]	Retrospective cohort design	Patients with T2DM and chronic lower respiratory disease newly initiated on a GLP-1RA (*n* = 4,150) or DDP-4I (*n* = 12,540)	↓ Hospitalization rates ↓ Respiratory exacerbations rates
Foer, et al. [91]	Retrospective, observational, electronic health records-based study	Patients with COPD with new prescriptions for DPP-4i (*n* = 260), GLP-1RA (*n* = 328), SGLT2i (*n* = 353), or sulfonylureas (*n* = 701)	↓ Exacerbation rates in GLP1RAs compared to DPP-4i ↓ Exacerbation rates in GLP1RAs compared to sulfonylureas
Pradhan, et al. [79]	Population based cohort study	Patients with COPD starting a GLP-1RA (*n* = 1252), DPP4-i (*n* = 8731), SGLT2i (*n* = 2956) or sulfonylurea	↓ Risk of moderate exacerbations compared to sulfonylureas ↓ Risk of severe exacerbations compared to sulfonylureas
Huang, et al. [73]	Cross-sectional study	Patients with COPD (*n* = 40) and matched non-COPD subjects (*n* = 35) provided peripheral blood samples	↓ Expression of GLP-1R on mononuclear cells in COPD patients at baseline ↑ IFN-γ production on mononuclear cells ↓ *PD-1* expression in CD4 and CD8 cells
Foer, et al. [8]	Retrospective electronic health records-based cohort study	Patients with T2DM and asthma newly initiated on GLP-1RA (*n* = 448), SGLT2i (*n* = 112), DPP-4i (*n* = 435), sulfonylureas (*n* = 2,253), or basal insulin (*n* = 2,692)	↓ Asthma exacerbations ↓ Healthcare encounters ↓ Asthma symptoms (except compared to SGLT2i)
Wang, et al. [77]	Retrospective electronic health records cohort study	Patients with T2DM and asthma comparing those initiated on GLP1RA (*n* = 7,939) vs. thiazolidinediones (*n* = 3,050) and GLP1RAs (*n* = 6,084) vs. sulfonylureas (*n* = 11,004)	↓ Exacerbations compared to sulfonylurea ↓ Exacerbations compared to thiazolidinediones, but did not reach significance
Khan, et al. [10]	Observational prospective cohort study	Patients with asthma and T2DM (*n* = 9) initiated GLP-1RA for 52 weeks	↓ Mild/moderate asthma exacerbations ↓ Mean ACQ in those who lost >2.9 kg
See et al. [92]	Retrospective cohort study	Patients with COPD and T2DM (*n* = 6896) initiated on GLP-1RA (*n* = 4184) or DPP4 inhibitor (1751)	↓ COPD exacerbations ↓ Risk of oxygen dependence ↓ All-cause mortality
Kaplan et al. [93]	Retrospective cohort study	Patients with asthma and obesity initiated on GLP-1RAs (*n* = 10,111) and unexposed controls (*n* = 50,555)	↓ BMI (~ 7.7 pounds) ↑ Overall asthma control ↑ Risk domain asthma control No significant difference in asthma exacerbations
Wang et al. [101]	Network meta-analysis	Patients with asthma with or without T2DM (*n* = 159,705) receiving a GLP-1RA (*n* = 55,973), DPP4-i (*n* = 47,714), SGLT2i (*n* = 56,018)	↓ Exacerbation rates SGLT2i compared to placebo No significant difference in GLP-1RA groups
Foer et al. [100]	Retrospective cohort study	Patients with asthma and T2DM prescribed GLP-1RAs (*N* = 40) or comparator therapies (*n* = 110)	↓ Serum periostin No significant differences in IgE, IL-6, IL-8, sCD163
Hogan et al. [95]	Small prospective interventional study	Patients with T2DM (*n* = 10) started on GLP-1RA (Liraglutide), results compared to baseline	↓ Pro-inflammatory cytokines: TNF-α, IL-1β, IL-6 ↓ Anti-inflammatory adipokine adiponectin ↓ Inflammatory macrophage activation marker, sCD163
Bray et al. [96]	Systematic Review and meta-analysis	Patients with T2DM receiving GLP-1RA, standard diabetic therapies, or placebo with age/sex matched lean non-diabetic controls	↓ Inflammatory biomarkers: CRP, TNF-α ↓ Inflammatory oxidative stress biomarkers: MDA ↑ Adiponectin
*Retrospective Controlled Trials*			
Atlantis, et al. [80]	Randomized double blind controlled trial	Patients with obesity (BMI >27) and COPD allocated GLP-1RA (*n* = 20) or placebo (*n* = 20)	↑ weight loss ↑ FVC and DLCO at Week 40 ↓ CAT score at Week 40
Lopez-Cano, et al. [81]	Randomized double blind crossover-controlled trial in diabetes looking at lung function	Patients with T2DM (*n* = 76) administered GLP-1RA followed by placebo or vice versa	↑ FVC ↓ Serum surfactant protein D ↓ Serum surfactant correlated with ↑ FVC

T2DM: Type 2 diabetes mellitus. OSA: Obstructive Sleep Apnea. CPAP: continuous positive airway pressure. BMI: Body mass index. AHI: apnea hypopnea index. hsCRP: high-sensitivity C-reactive protein. LPS: lipopolysaccharide. GLP-1RA: glucagon-like peptide-1 receptor agonist. DDP-4I: dipeptidyl-peptidase 4 inhibitor. SGLT2: sodium-glucose cotransporter 2. CD69: cluster of differentiation 69. FVC: forced vital capacity. FEV: forced expiratory volume. DLCO: carbon monoxide diffusion capacity. CAT: COPD assessment test. IFN-γ: interferon gamma. PF-1: programmed cell death protein-1. ACQ: asthma control questionnaire. COPD: chronic obstructive pulmonary disorder. MEF: Maximal expiratory flow. TLC: total lung compacity. RV: residual volume. MCP-1: monocyte chemoattractant protein-1. NF-κB: nuclear factor kappa-light-chain-enhancer of activated B cells. MDA: serum malondialdehyde. IL: interleukin. TNF-α: tumor necrosis factor-alpha. IL-1β: interleukin-1 beta. HR: hazard ratio. CL: confidence limit. OR: odds ratio

### Metabolic Improvements and Weight loss / Role of GLP-1 in Metabolic Regulation/Systemic Regulation

Large retrospective studies involving 448 and 9134 patients with asthma and T2DM, found that individuals prescribed GLP-1RAs experience significantly lower rates of asthma exacerbations, nearly two-fold lower compared to those on other diabetic agents [8, 77]. Furthermore, prospective cohort studies have demonstrated that GLP-1RA treatment leads to improvements in forced expiratory volume in one second (FEV_1_), forced vital capacity (FVC), and maximal expiratory flow (MEF) compared to baseline, insulin-treated, and control cohorts [78]. Retrospective studies demonstrated reduced COPD exacerbations in T2DM patients on GLP-1RAs, as compared to DPP-4 inhibitors and sulfonylureas [9, 79]. Similarly, in a double-blind controlled trial among patients with obesity and COPD revealed that liraglutide resulted in increased FVC, carbon monoxide diffusion capacity (DLCO), and COPD assessment test (CAT) scores. However, there were no significant changes in FEV_1_ or FEV_1_/FVC ratio [80]. These findings suggest that the benefits of GLP-1RAs could potentially extend beyond glycemic control.

### Weight Loss

One of the most notable benefits of GLP-1RAs is their ability to induce significant weight loss, which has implications for both diabetes management and lung function. Weight loss demonstrates improvement in quality of life, asthma control, and lung function in patients with pulmonary diseases and obesity [81]. Indirectly, significant weight loss may not only diminish obesity-induced inflammation, but it also reduces obesity-related mechanical constraints on the diaphragm and chest wall, improves expiratory reserve volume (ERV) on pulmonary function testing, and reduce restrictive physiology that can be observed in individuals that are obese and have asthma. Increased adiposity is linked to decreased lung function possibly with asthma-like obstructive changes or restrictive deficits due to thoracic cage impedance [82, 83]. This is observed in people who undergo bariatric surgery where significant weight loss is accompanied by improvements in pulmonary function parameters such as FEV_1_, FVC, total lung capacity (TLC), and DLCO [84, 85].

GLP-RA treatment is associated with weight loss that may lead to pulmonary improvement. A small prospective observational study of individuals with asthma who were prescribed liraglutide for T2DM, found that those who lost more than 6.4 pounds experienced improvements in their asthma control questionnaire (ACQ) scores [10]. Randomized controlled trials investigating the effects of liraglutide on T2DM patients with OSA, a condition that often overlaps with individuals with obesity and asthma, demonstrated significant weight loss and improvements in respiratory function. After three months, the liraglutide group showed reduced BMI, significantly improved apnea-hypopnea index (AHI), and higher oxygen saturation, indicating enhanced pulmonary function. These findings were noted both in the presence and absence of continuous positive airway pressure (CPAP) therapy [86–89]. The GLP-1RA, tirzepatide, also reduced hypoxic burden and high-sensitivity C-reactive protein (hsCRP) in patients with moderate-to-severe OSA and obesity [88].

### Independent of Weight Loss (Humans)

While weight loss plays a key role in improving pulmonary outcomes with GLP-1RAs, recent studies that account for BMI suggest that the benefits extend beyond metabolic changes. Liraglutide therapy in patients with T2DM, obesity, and have asthma led to significantly fewer asthma exacerbations [10]. Amin et al. also noted improvement in AHI, independent of weight loss, indicating other possible mechanisms [90]. Foer and colleagues found that the reduced asthma exacerbations in the GLP-1RA treated group were independent of changes in hemoglobin A1C or BMI [8]. López-Cano and colleagues conducted a double-blind, randomized, crossover, placebo-controlled clinical trial investigating the effects of liraglutide over a 7-week period, while minimizing weight loss as a confounding factor. They observed improvements in FVC and a significant reduction in serum surfactant protein D (SP-D) levels, a lung-specific biomarker for inflammation that functions as an endogenous airway anti-microbial and anti-inflammatory molecule [81]. This reduced risk of acute exacerbations and pulmonary-related mortality is also seen in patients with COPD [91, 92]. Moreover, a small prospective observational study found that patients treated with GLP-1RA experienced significant improvements in asthma outcomes, including fewer hospitalizations, reduced need for oral corticosteroids, and fewer short acting β2AR agonist (SABA) prescriptions compared to non-GLP-1RA treated patients. These benefits were observed even after adjusting for BMI and corticosteroid use, reinforcing the idea that GLP-1RAs may independently improve lung function and have a protective or anti-inflammatory role in pulmonary diseases beyond weight loss [93].

### Regulation of Inflammatory and Immune Responses by GLP-1 Receptor Agonists

Research into the lung-protective effects of GLP-1RAs has expanded in recent years, with evidence suggesting potential mechanisms linked to their anti-inflammatory properties [94]. In an observational study, individuals that have T2DM, GLP-1RA therapy, Liraglutide, reduced pro-inflammatory cytokines (e.g., TNF-α, interleukin-1 beta (IL-1β), IL-6) in PBMCs, and increased serum adipokine, adiponectin, and macrophage activation markers (sCD163) [95]. GLP-1RAs could potentially skew the human macrophage phenotype toward an anti-inflammatory state by increasing IL-10 and suppressing pro-inflammatory cytokines such as TNF-α, IL-1β. This immunomodulatory effect may contribute to their broader anti-inflammatory actions [71].

A systematic review and meta-analysis by Bray and colleagues assessed the effects of GLP-1RAs on biomarkers of inflammation and oxidative stress in individuals with T2DM, and reported a significant reduction in CRP, TNF-α, malondialdehyde, along with significant increases in adiponectin levels [96]. In a randomized, double-blind, placebo-controlled trial, liraglutide significantly decreased the proinflammatory cytokine IL-6 with similar trends for other proinflammatory cytokines in humans [97]. Moreover, in a randomized study with T2DM patients, liraglutide combined with insulin significantly improved glycemic control, reduced oxidative stress markers, increased antioxidant levels, and lowered pro-inflammatory markers, MCP-1 and nuclear factor kappa-light-chain-enhancer of activated B cells (NF-kB), as compared to insulin alone [98].

GLP-1 analogues may also ameliorate COPD via augmenting normal T-cell functions where T-cell dysregulation is known to contribute to the progression of COPD [99]. An observational study in patients with COPD showed that GLP-1 receptor expression in circulating PBMCs was decreased compared with non-COPD patients, resulting in dysfunctional interferon gamma production and upregulation in programmed cell death 1 (PD-1) in both CD4 + and CD8 + T cells. These findings were attenuated by liraglutide therapy, demonstrating potential T-cell regulation by GLP-1 RAs in COPD [73].

In contrast, studies of GLP-1RAs directed specifically at understanding their impact in pulmonary diseases in patients without obesity or T2DM remain limited. This is largely due to their FDA-approved indications for use in T2DM and obesity only. Foer and colleagues aimed to fill that gap by examining serum periostin, a biomarker strongly associated with airway inflammation induced by IL-4, IL-13, and transforming growth factor-β [100]. They found that serum periostin levels significantly decreased in T2DM patients treated with GLP-1RAs, independent of weight loss compared to non-GLP-1RA cohort [100]. Although no significant differences were observed in other inflammatory markers like IgE, IL-6, IL-8, or soluble CD164 (sCD164). This aligns with findings from studies like Dogan et al., where liraglutide improved lung function in COPD patients without decreasing systemic inflammation markers such as CRP, IL-5, or monocyte chemoattractant protein-1 (MCP-1) [80]. This absence of inflammation reduction may be attributed to the mild severity of COPD in their cohort. Despite these findings, large-scale clinical evidence for GLP-1RAs in asthma remains inconclusive. A network meta-analysis by Wang et al. found that GLP-1RAs did not significantly reduce asthma exacerbations, whereas, SGLT-2 inhibitors (SGLT-2i) were associated with a reduced risk of asthma compared to both GLP-1RAs and DPP-4 inhibitors (DPP-4i) [101]. However, the low incidence rate of asthma events in these trials limits the impact of these conclusions. Given asthma’s immunological complexity and heterogeneous inflammatory endotypes, further mechanistic and long-term studies including randomized clinical trials are warranted to clarify the role of GLP-1RAs in asthma management.

## Pre-Clinical Animal Studies

### GLP-1RA and Pulmonary Inflammation

Animal models are advantageous in exploring the mechanistic effects of GLP-1 on pulmonary function and disease. In murine models of lung injury, the administration of exogenous GLP-1RAs confers significant protection against pulmonary inflammation and airway hyperresponsiveness. These protective effects are mediated, in part, through modulation of key inflammatory pathways central to asthma pathophysiology, summarized in Table 2. For instance, GLP-1 receptor knockout (*Glp1r*^–/–^) mice of C57BL/6 background exhibit significantly worse pulmonary inflammation following an influenza viral infection, indicating a protective role of GLP-1 in modulating immune responses in the lung [102].

**Table 2:** In vitro studies using collected human specimens

Reference	Study	Population	Main Findings (Comparing GLP-1 users to non-GLP-1 users)
Buldak et al. [71]	Experimental in-vitro human study	Monocytes/macrophages harvested from 10 healthy subjects treated with GLP-1RA (Exenatide), GLP-1 receptor antagonist (Exendin 9–39), or vehicle	↓ TNF-α, IL-1β ↑ IL-10 in culture medium ↑ mRNA expression of *Arg1* ↓ *iNOS* mRNA expression and protein concentration
Rogliani, et al. [75]	Ex vivo lab study	Human isolated bronchial tissue incubated in glucose concentrations and passively sensitized in serum, followed by electrical field stimulation and GLP-1 administration	↓ Electrical field induced contractile tone ↓ Bronchial hyperresponsiveness in high glucose stimulation and sensitization

GLP-1RA: Glucagon-like peptide-1 receptor agonist. TNF-α: Tumor necrosis factor alpha. IL-1β: Interleukin-1 beta. IL-10: Interleukin-10. Arg1: Arginase 1. iNOS: Inducible nitric oxide synthase

**Table 3:** Mouse studies

References	Study	Main Findings
Toki et al. [65]	Female BALB/c mice aged 9–12 weeks challenged with Alternaria extract or house dust mite; GLP-1R agonist (Liraglutide) or vehicle	Alternaria Extract: ↓ IL-33 release ↓ ILC2 expressing IL-5 and IL-13 ↓ T2 cytokines and chemokines in BALF: IL-5, IL-13, CCL17, CCL22, CCL24 ↓ T2 cytokines in lung homogenate: IL-4, IL-5, IL-9, IL-13, CCL11, CCL17, CCL22, CCL2 ↓ BALF inflammatory cell counts ↓ Mucin production ↓ Airway hyperresponsiveness ↓ *Duox1* mRNA expression House Dust Mite: ↓ Inflammatory mediators in BALF: IL-4, IL-5, IL-13, CCL11, CCL17, CCL22, CCL24 ↓ Inflammatory mediators in lung homogenate: IL-4, IL-5, IL-13, IL-9, CCL11, CCL17, CCL22, CCL24 ↓ BALF Inflammatory cell counts
Zhang et al. [66]	Male Sprague-Dawley rats aged 10–12 weeks; GLP-1RA (Liraglutide) or vehicle	↑ Pulmonary function ↓ Lung injury ↓ Inflammatory mediators of perfusate: IL-6, TNF-α, IL-1β, IL-18, IL-10, CXCL2)
Sato et al. [102]	Male wildtype *Glp1r*^+/+^ or *Glp1r*^–/–^ mice aged 9 to 10 weeks; bleomycin hydrochloride or influenza virus model; GLP-1R agonist (Liraglutide) or vehicle	Non-infectious model: ↓ Lung injury with GLP-1R loss ↑ Pulmonary inflammation with GLP-1R activation Infectious model: ↓ Pulmonary inflammation with GLP-1R agonism ↓ Virus load with GLP-1R agonism
Zhu et al. [103]	Male BALB/C mice aged 6–8 weeks; Ovalbumin or PBS; GLP-1RA (Liraglutide) or vehicle	↓ BALF Inflammatory cell counts ↓ Inflammatory Mediators in BALF: TNF-α, IL-4, IL-5, IL-13 ↓ Airway mucin secretion and goblet cell hyperplasia ↓ NF-κB p65 DNA binding activity and activation, phosphorylation of PKA ↓ E-selectin, MUC5AC
Toki et al. [104]	SWR (lean) and TALLYHO (obese) mice; aeroallergen or PBS; GLP-1RA (Liraglutide) or vehicle	↓ Inflammatory mediators in BALF: IL-5, IL-13, CCL11, CCL24, CXCL1, CXCL5, IL-1β ↓ Airway neutrophil and eosinophil recruitment ↓ Airway responsiveness of TALYHO mice ↓ BALF TSLP and IL-33 in TALLYH mice ↓ Lung ILC2 proliferation in TALLYHO mice
Bloodworth et al. [105]	Female mice aged 8 weeks infected with virus-associated lung inflammation; GLP-1RA (Liraglutide) or vehicle	↓ Inflammatory mediators in lung homogenate: IL-13, IL-33 ↓ IL-13^+^ ILC2s, IL-13^+^ Th2, IL-13^+^ basophils ↓ Total lung cells ↓ Mucin secretion ↓ Airway responsiveness
Toki et al. [107]	Female mice aged 9–13 weeks (WT, GLP-1R KO, GIPR KO, GLP-1R/GIPR DKO) challenged with Alternaria extract; GLP-1R agonist (Tirzepatide) or vehicle	Alt-Ext challenged GLP-1R/GIPR DKO model: ↑ TSLP and HMGB1 in BALF ↑ Inflammatory mediators in lung homogenate: IL-5, IL-13, CCL11, CCL24) ↑ Lung ICL2 cells in lung homogenate ↑ ICAM-1 expression With Tirzepatide Treatment: ↓ BALF inflammatory cell counts in WT mice ↓ Inflammatory mediators in the lung homogenate: IL-5, IL-33, CCL24 in WT mice No anti-inflammatory effect in GLP-1R KO or GLP-1R/GIPR DKO mice
Toki et al. [108]	Female polygenic obese TALLYHO mice challenged with Alt-Ext; GIPR agonist, Semaglutide, Tirzepatide or vehicle	With Tirzepatide Treatment: ↓ Inflammatory mediators in lung homogenate: IL-5, IL-6, IL-13, IL-33, CCL11, CCL17, CCL22, CCL24 ↓ BALF inflammatory cell counts ↓ Lung CD4^+^ T cells and Th2 cells With Semaglutide Treatment: ↓ Inflammatory mediators in lung homogenate (IL-6, IL-33, CCL11, CCL17, CCL22, CCL24) ↓ BALF inflammatory cell counts ↓ Lung CD4^+^ T cells
Wong et al. [115]	Male and female C57BL/6J mice subjected to polymicrobial sepsis; treated with GLP-1RA (Exendin-4) or vehicle; with neuronal-specific GLP-1R KO models used for mechanistic insight	↓ Plasma TNF-α induced by LPS ↓ Blood leukocyte and lung *Tnf* expression ↓ Inflammation via central neuronal GLP-1Rs ↓ Plasma cytokines and chemokines: TNF-α, IL-1β, IL-6, CXCL1
Baer et al. [116]	Male and female C57BL/6 mice aged 8–12 weeks; induced polymicrobial abdominal sepsis or control; pretreated with GLP-1RA (Liraglutide) or vehicle	↓ BALF total cell counts ↓ Inflammatory mediators in BALF: TNF-α, IL-6, IL-1β, CXCL-1 ↓ Alveolar-capillary barrier disruption ↓ Histological lung injury ↓ RAGE content ↓ Alveolar-capillary barrier disruption ↓ *iNOS* mRNA expression

BALF: bronchoalveolar lavage fluid. CD11b: cluster of differentiation 11b. CD69: cluster of differentiation 69. COPD: chronic obstructive pulmonary disease. CXCL1: C-X-C motif chemokine ligand (1) CXCL2: C-X-C motif chemokine ligand (2) CXCL5: C-X-C motif chemokine ligand 5. Duox1: dual oxidase 1. GLP-1RA: glucagon-like peptide-1 receptor agonist. GLP-1R: glucagon-like peptide-1 receptor. GIPR: glucose-dependent insulinotropic polypeptide. GAGs: glycosaminoglycans. iNOS: inducible nitric oxide synthase. ILC2: group 2 innate lymphoid cells. IL-1β: interleukin-1 beta. IL-4: interleukin-4. IL-5: interleukin-5. IL-6: interleukin-6. IL-8: interleukin-8. IL-9: interleukin-9. IL-10: interleukin-10. IL-13: interleukin-13. IL-18: interleukin-18. IL-33: interleukin-33. LPS: lipopolysaccharide. MUC5AC: mucin 5AC. NF-κB: nuclear factor kappa-light-chain-enhancer of activated B cells. OVA: ovalbumin. PBS: phosphate-buffered saline. PKA: protein kinase A. PPAR-γ: peroxisome proliferator-activated receptor gamma. PTEN: phosphatase and tensin homolog. RAGE: receptor for advanced glycation end-products. TARC (CCL17): thymus and activation-regulated chemokine. T2: type 2 (immune response). TNF-α: tumor necrosis factor alpha. TSLP: thymic stromal lymphopoietin

**Table 4:** Mechanistic pathways through which GLP-1 agonists improve T2 low asthma in relation to obesity

Section	Mechanistic Pathway	Key Effects / Mechanisms
1. Indirect effects through weight loss	Pulmonary function improvements	Improvements in FEV₁, FVC, and mid-expiratory flows; reductions in airway resistance; improved small airway function - most strongly correlated with decreases in truncal fat and waist circumference.
	Reduction in asthma comorbidities	A. Obstructive Sleep Apnea (OSA): i. Reduced upper airway and tongue adiposity ◊ less airway collapsibility. ii. Increased functional residual capacity ◊ enhanced upper airway traction. B. Gastro-esophageal reflux: improved control. C. Rhinitis: variable improvements.
2. Metabolic and inflammatory effects that decrease asthma risk	Reduction in systemic inflammation	Decreased IL-6, IL-8, TNF-α, CRP, and adipokines; shift toward anti-inflammatory state with increased IL-10.
	Metabolic improvements	Improved hyperinsulinemia and hyperglycemia, leading to better airway function and reduced metabolic stress on the respiratory system.
3. Direct effects on airway inflammation and smooth muscle function (Fig. 2)	Immune modulation and inflammatory suppression	↓ ILC2 → reduced IL-5 and IL-13 (key Th2 cytokines). Modulation of T-cell responses. Suppression of NF-κB via PKA-dependent pathway ◊ reduced macrophage activation and neutrophil recruitment. Downregulation of MUC5B gene expression.
	Chemokine and oxidative stress regulation	↓ Chemokines activating eosinophils (IL-9, CCL11, CCL17, CCL22, CCL24). ↓ Inducible NOS and DUOX1 ◊ reduced oxidative stress and epithelial damage. ↓ Advanced glycation end-products (AGEs).
	Airway smooth muscle and nitric oxide pathways	Activation of intracellular signaling leading to β₂AR-mediated airway smooth muscle relaxation. Restoration of arginine-mediated NO production and upregulation of eNOS → enhanced bronchodilation.

FEV₁: Forced Expiratory Volume in 1 s. FVC: Forced Vital Capacity. IL: Interleukin. ILC2:Type 2 Innate Lymphoid Cell. TNF-α:Tumor Necrosis Factor-alpha. CRP: C-reactive protein. NF-κB: Nuclear Factor kappa-light-chain-enhancer of activated B cells. PKA: Protein Kinase A. MUC5B: Mucin 5B gene. CCL: C-C motif chemokine ligand. NOS: Nitric Oxide Synthase. DUOX1:Dual Oxidase 1. AGEs: Advanced Glycation End-products. β₂AR: Beta-2 Adrenergic Receptor. NO: Nitric Oxide. eNOS: Endothelial Nitric Oxide Synthase

Treatment with GLP-1RAs reduced inflammatory cell populations, pro-inflammatory cytokines within BALF, and pro-inflammatory cytokines in lung homogenates across multiple allergen and injury models including *Alternaria*, house dust mite (HDM), and ovalbumin aeroallergen challenges, and bleomycin or virus-associated lung injury [65, 66, 102–108]. Studies using GLP-1R and GLP-1R/glucose-dependent insulinotropic polypeptide receptor (GIPR) knockout mice further show that loss of these receptors exaggerates allergen-induced airway inflammation, demonstrating that endogenous incretin receptor signaling normally limits type 2 innate immune responses [107]. These cytokines drive type 2 inflammatory responses and are closely linked to airway eosinophilia, mucin hypersecretion, and bronchial hyperreactivity. Notably, this anti-inflammatory effect extends to non-allergic models as well, where GLP-1 receptor activation reduces levels of IL-6, TNF-α, IL-1β, and CXCL2, while increasing markers such as IL-10 and arginase-1 [66, 71, 109–111]. Additional reductions are observed in inflammatory mediators including monocyte chemoattractant protein-1 (MCP-1), inducible nitric oxide (NO) synthase (iNOS), and NO production [66, 109, 110].

GLP-1RAs also downregulate chemokines such as IL-9, CCL11 (eotaxin-1), CCL17 (TARC), CCL22 (MDC), and CCL24 (eotaxin-2) in both BALF and lung tissue homogenates. These chemokines recruit and activate eosinophils, Th2 cells, and other immune effectors in an allergic airway response [65]. In vitro studies further demonstrate that GLP-1 analogs directly reduce human eosinophil activation as evidenced by decreased surface expression of CD69 and CD11b, and suppressed secretion of IL-4, IL-8, and IL-13 [70]. Similar effects on CD11b expression have been observed in neutrophils in vivo with GLP-1R blockade abolishing this response [112]. Beyond effects on inflammatory cells, GLP-1RAs also suppress group 2 innate lymphoid cells (ILC2) activity, significantly reducing their production of IL-5 and IL-13, cytokines critical for eosinophil survival and goblet cell metaplasia [65]. This is accompanied by broader reduction of type 2 inflammation, including decreased IL-13 and IL-33, and reduced frequencies of IL-13-producing ILC2, Th2 cells, and basophils in the lung [105]. Together, these findings suggest that GLP-1RAs dampen T2 immune responses through both direct and indirect effects on key inflammatory cell populations.

Consistent with these immunomodulatory effects, GLP-1 analogs also directly reduce mucin hypersecretion. In an ovalbumin-induced chronic asthma mouse model, liraglutide markedly inhibited the up-regulation of MUC5AC, leading to a decreased mucin hypersecretion [103]. This finding aligns with lixisenatide, a selective GLP-1RA, significantly reducing MUC5AC expression in LPS-treated human bronchial epithelial cells [113]. Fork head box protein A2 (FOXA2) is a key transcriptional regulator of airway mucin homeostasis in diseased airways and is under expressed in airways of people with COPD. The GLP-1 analog, exenatide, reduced mucin production by restoring FOXA2 function through the activation of GLP-1 receptor-peroxisome proliferator-activated receptor gamma (PPAR-γ)-phosphatase and tension homolog (PTEN) signaling in COPD-derived human primary bronchial epithelial cells [114]. Together, these findings highlight how GLP-1RAs not only modulate immune responses but also directly target epithelial mechanisms to reduce mucin overproduction in airway diseases.

### GLP-1RAs Acting Through the Gut-brain-lung Axis

GLP-1R activation attenuates systemic inflammation and TNF-α production, induced by multiple Toll-like receptor agonists, through the gut-brain axis, specifically via the central neuronal GLP-1Rs. This neuroimmune regulatory pathway was critical for reducing systemic responses and lung injury in a polymicrobial sepsis model and involved downstream α1-adrenergic and opioid receptor pathways [115]. Complementing these findings, a second study found that liraglutide pretreatment significantly mitigates lung injury in a murine two-hit model of sepsis-induced acute respiratory distress syndrome (ARDS). Liraglutide reduced inflammatory cell infiltration, cytokine release, epithelial injury, and alveolar-capillary barrier disruption, ultimately improving physiological outcomes [116]. These studies highlight the therapeutic potential of GLP-1RAs as systemic anti-inflammatory agents, acting through central and peripheral mechanisms.

### GLP-1RAs Acting Through cAMP/PKA/NFkB Signaling Pathways

The GLP-1 receptor functions as a stimulatory G-protein that activates the guanine nucleotide binding proteins (G proteins), specifically through the G protein alpha subunit group S (G_s_α) signaling cascade to increase cyclic adenosine monophosphate (cAMP) accumulation and activate protein kinase A (PKA). Mechanistically, GLP-1 carries out its various functions through a PKA-dependent signaling pathway, which plays a key role in regulating NF-κB activation across multiple inflammatory conditions [113, 117–120]. For example, GLP-1 increases PKA phosphorylation and simultaneously suppresses NF-κB DNA-binding activity in the lung, effects that are abolished by PKA inhibitor H-89. GLP-1RAs suppress lung inflammation and mucin hypersecretion through a PKA-dependent NF-κB signaling pathway [103]. In an experimental in vitro study, the anti-inflammatory effects of GLP-1RA, exenatide, on human macrophages are predominantly mediated through PKA signaling [71]. GLP-1RAs also reduce macrophage activation and TNF-α expression in vivo through the PKA activation and NF-κB inhibition in an LPS-induced allergic mouse model [121]. Similarly, in vitro studies demonstrated that GLP-1RA reduced IL-1β production by pro-inflammatory M1 macrophages and increase of IL-10 production by anti-inflammatory M2 macrophages [95]. These findings support a role for GLP-1R signaling in modulating macrophage polarization, contributing to its overall anti-inflammatory effects.

GLP-1 receptors regulate intracellular pathways like those activated by β2AR agonists, that are responsible for ASM relaxation in asthma. GLP-1 receptor agonists have bronchodilator effects [78] through their role as a GPCR-activating cAMP/PKA cascade, leading to ASM relaxation [75]. As a result, combining a GLP-1 receptor agonist with an anti-muscarinic agent may result in an additional or synergistic broncho-relaxant effects due to complementary mechanisms of action, as seen with different classes of bronchodilators administered in combination [122]. A recent ex vivo study using isolated human bronchi demonstrated that treatment with the GLP-1RA, exendin-4, modulated bronchial contractile tone and significantly reduced bronchial hyperresponsiveness and airway inflammation induced by high-glucose conditions and passive sensitization [75].

### GLP-1RAs Modulation on Arginine-Nitric Oxide Pathways and AGE-RAGE Signaling

Arginine metabolism plays a crucial role in regulating NO bioavailability, a key mediator of endothelial and epithelial function in the lung. The pathway is finely tuned by both nitric oxide synthase (NOS) and asymmetric dimethylarginine (ADMA), an endogenous inhibitor of NOS. In diseases like asthma and ARDS, dysregulated arginine metabolism characterized by increased ADMA levels and reduced NO production, leads to epithelial dysfunction and increased oxidative stress. This imbalance contributes to airway inflammation, airway hyperreactivity, and epithelial injury, which are hallmarks of airway disorders. Arginine supplementation increases NO levels, resulting in bronchodilation and a reduction in inflammation in moderate to severe persistent asthma patients [123]. In addition, citrulline, a major precursor for endogenous arginine, supplementation has been shown to restore systemic arginine levels, improve airway NO production, and asthma control in individuals with obesity-related asthma [124]. Interestingly, arginine supplementation can stimulate GLP-1 secretion in diet-induced obese mice [125].

Recent studies have highlighted the potential of GLP-1RAs in restoring arginine-mediated NO production. GLP-1RAs downregulate inducible NOS (iNOS) and dual oxidase 1 (DUOX1), both of which contribute to oxidative stress and epithelial damage [65, 116, 126]. GLP-1 can elevate NO production through upregulation of endothelial NOS (eNOS), a response that is abolished by eNOS inhibition [127]. Similarly, in a rat model of metabolic syndrome, GLP-1RA increased NO production and reduced oxidative stress [128]. By attenuating iNOS and shifting arginine metabolism toward NO bioavailability and reduced ADMA levels, GLP-1RAs can enhance epithelial barrier function and mitigate ROS generation.

**Fig. 2:**
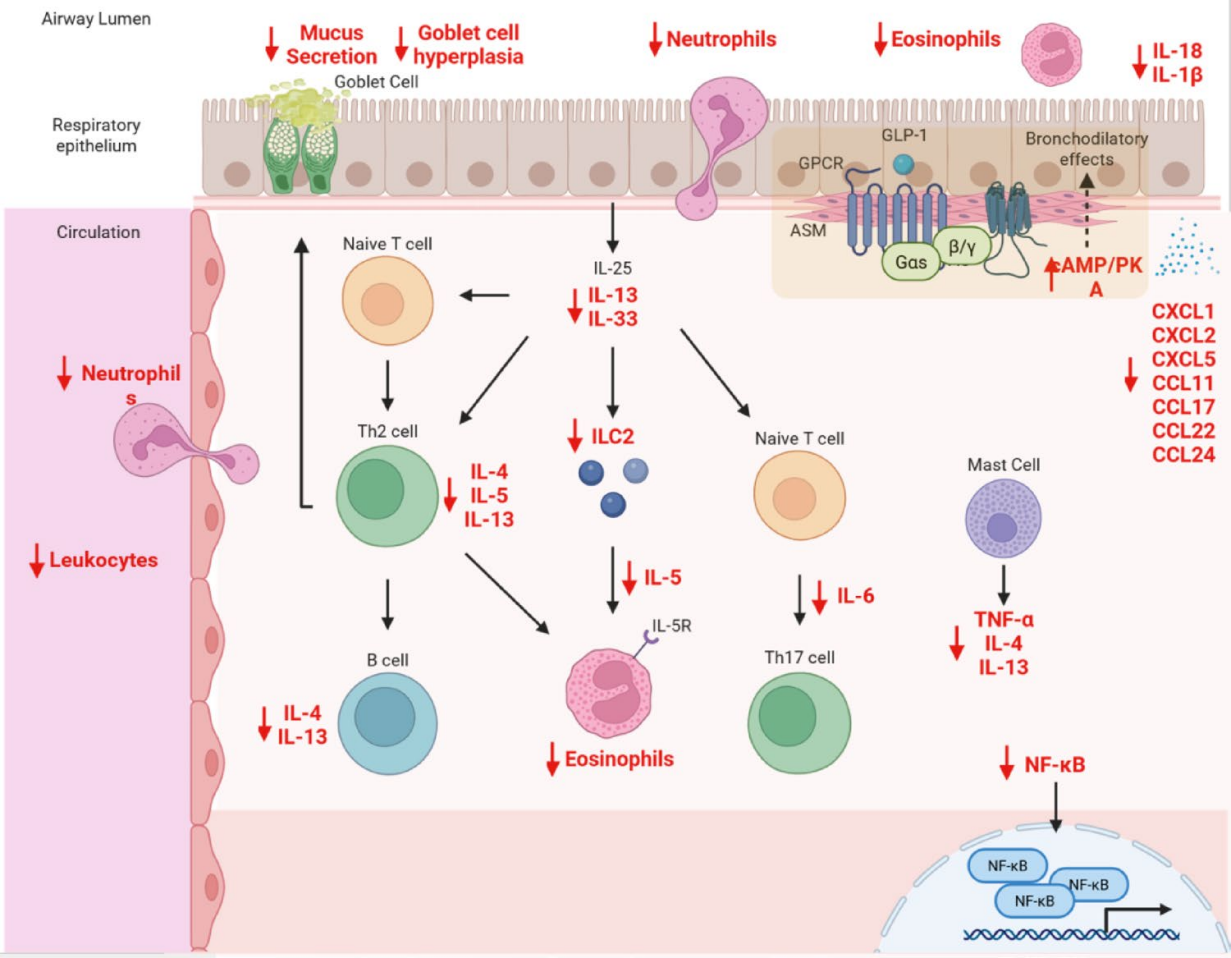
Potential mechanisms as to how exogenous GLP-1 is acting on the lung

In addition to their effects on NO, GLP-1RAs also exert antioxidant effects. These effects are crucial in diseases like asthma, where oxidative damage is a major contributor to airway remodeling [129]. GLP-1RAs reduce the accumulation of advanced glycation end-products (AGEs), which bind to the receptor for AGEs (RAGE), and trigger pro-inflammatory pathways that contribute to lung pathology [130–134]. AGEs can suppress NO bioavailability [135], which is impaired in allergic airway disease [136]. GLP-1 has been shown to inhibit RAGE and decrease inflammation in human retinal pigment epithelium [137]. Similarly, GLP-1RAs can inhibit the AGE-RAGE-ROS pathway, halting the progression of diabetic nephropathy [138]. Although these effects have been primarily studied in other organs, such mechanisms may also be relevant to the lung. Supporting this, GLP-1RA attenuates sepsis-induced alveolar epithelial injury by reducing BALF RAGE levels and glycocalyx shedding, key markers of alveolar-capillary barrier dysfunction in ARDS [116]. Collectively, these findings suggest GLP-1RAs may serve as dual-purpose agents by targeting both metabolic and inflammatory components of asthma by modulating arginine metabolism, reducing oxidative stress, preserving lung epithelial integrity, and counteracting inflammation-driven lung injury, with potential mechanistic insight shown in Fig. 2.

### Future Directions, Current Limitations, and Emerging Areas for Research

In conclusion, the collective clinical and preclinical evidence robustly supports the potential of GLP-1RAs as a promising therapeutic strategy for obesity-related asthma and/or low T2 asthma. Clinical studies demonstrate that GLP-1RAs not only improve metabolic parameters but also significantly reduce airway inflammation and improve lung function in individuals that are obese and diagnosed with asthma. Complementary findings from mouse models further elucidate the underlying mechanisms, revealing that GLP-1RAs modulate immune responses and decrease AHR. These dual benefits address both the metabolic dysfunction and pulmonary pathology that characterize obesity-related asthma. Future research should prioritize mechanistic studies using knockout and CRISPR-based models to precisely define the cellular pathways through which GLP-1RAs exert their anti-inflammatory and metabolic effects in the lung.

Despite these advances, additional clinical trials are needed to establish the efficacy, optimal dosing, and long-term safety of GLP-1RAs in obesity-related asthma. One such effort is the ongoing Glucagon-Like Peptide-1 Receptor Agonist in the Treatment of Adult, Obesity-related, Symptomatic Asthma (GATA-3) trial at Vanderbilt University Medical Center, which is evaluating semaglutide in adults with obesity and asthma who do not have diabetes. This randomized, placebo-controlled study is designed to provide clinical insight into how GLP-1RAs modulate airway inflammation and metabolic function in obesity-related asthma. Results from this and future studies will be essential for determining the role of GLP-1RAs in routine clinical management and for guiding the development of targeted, metabolism-informed asthma therapies.

## Conclusions

GLP-1RAs provide a novel approach for treating obesity-related asthma by simultaneously targeting metabolic dysfunction and airway inflammation. By addressing the complex interplay between metabolic and immunologic pathways, GLP-1RAs offer a comprehensive strategy to improve lung function, reduce exacerbations, and enhance overall patient outcomes. The growing preclinical and early clinical evidence supports their potential as a transformative therapy in this population.

## Data Availability

No datasets were generated or analysed during the current study.
